# Perceptions of COVID-19 risk during the pandemic: perspectives from people seeking medication for opioid use disorder

**DOI:** 10.1080/07853890.2023.2169342

**Published:** 2023-01-24

**Authors:** Sarah E. Clingan, Sarah J. Cousins, Chunqing Lin, Tram E. Nguyen, Yih-Ing Hser, Larissa J. Mooney

**Affiliations:** a Department of Psychiatry and Biobehavioral Sciences, University of California, Los Angeles, Los Angeles, CA, USA; b Department of Community Health Sciences, University of California, Los Angeles, Los Angeles, CA, USA; c VA Greater Los Angeles Healthcare System, Los Angeles, CA, USA

**Keywords:** Opioid use disorder, COVID-19, qualitative study, substance use, MOUD

## Abstract

**Introduction:**

The Coronavirus Disease 2019 (COVID-19) pandemic has had devastating consequences for persons with opioid use disorder (OUD). Yet, little is known about how people seeking treatment for OUD perceive the risks of COVID-19 and how their perception interplays with their health behaviours.

**Methods:**

In-depth interviews were conducted from September 2021 to March 2022 with 32 patients seeking medication treatment for OUD (MOUD) in Southern California. All interviews were conducted virtually and lasted between one and two hours. Interviews were recorded and transcribed verbatim. Two qualitative researchers independently conducted a content analysis of the transcripts to identify themes.

**Results:**

Three primary themes were identified: (1) perceptions and beliefs about COVID-19 susceptibility and severity; (2) perceptions of COVID-19 risk compared to substance use behaviours; and (3) vaccine hesitancy. Participants were mixed in their beliefs of susceptibility to contracting COVID-19 and the severity of the disease if contracted. Some participants reported taking precautions to mitigate their chances of acquiring COVID-19, and other participants reported that COVID was not a big concern as substance use took priority. For many of the participants, COVID-19 concerns were overshadowed by the risk of overdosing on substances and other risky substance use behaviour. Most of the participants (*n* = 23; 72%) had received at least one COVID-19 vaccine by the time of the interview, but over half (*n* = 19; 59%) expressed vaccine hesitancy. Vaccine hesitancy was driven by concerns about the unknown long-term side effects and potential interactions of the vaccine with MOUD.

**Conclusions:**

Our study provides insight into COVID-19 prevention measures as well as vaccination perceptions and hesitancy among people who received treatment for OUD.

**Key messages**
- Participants expressed diverse perceptions of the seriousness of COVID-19, with some taking precautions to mitigate their chances of acquiring COVID-19 and others perceiving that the risk of contracting COVID-19 was less than the risk of overdosing.
- Substance use, social isolation, vaccine hesitancy and COVID-19 risk behaviours should be studied as co-occurring phenomena that have potentially overlapping relationships that can influence behaviours that impact health and well-being.

## Introduction

The Coronavirus disease 2019 (COVID-19) pandemic has had devastating consequences for persons with opioid use disorder (OUD). Overdose deaths in the United States reached an all-time high during the pandemic, with provisional data from the Centers for Disease Control and Prevention estimating over 100,000 lives lost in the 12-month period ending April 2021 [1]. These numbers represent a 28.5% increase in overdose deaths from the same time period the year prior [2]. The rise in overdose deaths has been attributed to disruptions in treatment [3,4], increased stress from social isolation [5,6] and changes in drug use patterns [7]. The degree of treatment disruption during COVID is indicated by the 23% decrease in 2020 substance use disorder (SUD) treatment admissions from 2019 levels. Note, the largest reduction in the rates of treatment admissions was found among Native American and Black/African Americans [8]. Therefore, the interactions between the pandemic, stress and substance use are important factors contributing to the health of people with SUDs [9].

Individuals with SUDs and those who are at risk for SUDs are particularly vulnerable to the negative effects of COVID-19 [10]. Individuals with SUDs are more likely to contract the disease, are more likely to have negative health outcomes from the disease (e.g. death, hospitalization) [11] and are at increased risk for breakthrough infections than the general population [12]. Specifically, persons with a recent OUD diagnosis had a higher risk of COVID-19 diagnosis, hospitalization, or death compared to individuals with other SUDs or no SUD [11]. Comorbid health conditions [13] and unsafe living conditions [14,15] may increase the risk of contracting COVID-19 among people with OUD, compounding the risk attendant with vaccine hesitancy among this vulnerable population.

Vaccination against COVID-19 reduces the spread of the virus and lessens the severity of symptoms or post-condition complications (e.g. long-COVID) [16]. Yet vaccine hesitancy among individuals with SUDs is high [17]. A recent study showed that only 39.5% of individuals with SUDs in a residential treatment program trusted that a COVID-19 vaccine would be safe and effective. Thus, 60.5% were vaccine hesitant [17]. In comparison, a study conducted in 2020 found that 42.4% of U.S. adults were hesitant to obtain the vaccine [18]. This highlights the disparity in vaccine hesitancy among individuals with SUDs compared to the general population. Vaccine hesitancy tends to be higher among Black/African Americans and among individuals with lower income, whereas individuals of higher education levels and men have higher rates of vaccine acceptance [19]. Moreover, the same populations that are hesitant to vaccinate against COVID-19 also tend to experience severe consequences of OUD such as greater job loss, stigma, multiple morbidities, higher mortality rates, etc [20].

Pandemic-related mitigation measures to reduce the impact of COVID-19 resulted in temporary stay-at-home orders and closing of businesses. In turn, many individuals experienced reduced access to health and social services [21,22] and social and economic consequences (i.e. job loss and loss of social support systems, reduced access to healthcare). Pandemic-related stress compounds the overdose crisis in the United States, as stress is strongly linked to craving [23–25], higher severity of SUD and worse SUD treatment outcomes [26]. Likewise, loneliness and social isolation are strongly linked to increased stress levels and are associated with mental health problems including anxiety, depression and substance use [27–29].

The objectives of this study were to add to the existing literature on the links between substance use and COVID-19 by 1) exploring possible mechanisms by which substance use may exacerbate COVID-19 risk and 2) by examining interactions between these conditions in the period after COVID-19 vaccination was broadly available. We examined these phenomena by analysing data obtained from interviews with 32 patients who had received treatment for OUD in several clinics in three Southern California counties.

## Methods

Data were gathered from 32 patients from September 2021 to March 2022 who sought medication treatment for OUD (MOUD) at treatment sites (*N* = 15 sites that included outpatient SUD specialty treatment centres, a federally qualified health centre and MOUD treatment providers) that participated in the parent study, *Patient Decision Aid for Medication-Assisted Treatment* (‘PtDA study’; NCT03394261). The PtDA study was conducted in three large counties in Southern California to improve short-and long-term outcomes of patients who viewed a patient decision aid to assist them in making informed decisions about MOUD [30]. The current study conducted qualitative interviews with patients to better understand the relationship between substance use, COVID-19 risk and vaccination hesitancy during the COVID-19 pandemic when vaccines were accessible [31].

### Recruitment

PtDA study participants who indicated that they were interested in future research opportunities were recruited to participate in this study. A research associate contacted participants using an IRB-approved recruitment script to inquire about PtDA study participants’ interest in participating in an interview. Participants were eligible for this study if they were at least 18 years old and had sought MOUD treatment at a site that was involved in the PtDA study. Not all patients were active in MOUD treatment during the time they were interviewed (see Table 1). After the research associate verified eligibility and determined a mutually agreeable time to meet with the qualitative interviewer, potential participants were emailed an information sheet that described the study (e.g. purpose, participation, voluntary nature, confidentiality, risks, benefits and compensation for time) and an invitation to participate in a one-time virtual interview conducted *via* Zoom.

**Table 1.** Demographic data of study population (*N* = 32).

	N (%)
Sex	
Female	11 (34%)
Male	21 (66%)
Age (year)	
18–29	7 (22%)
30–49	17 (53%)
50–64	7 (22%)
65+	1 (3%)
Race	
White	19 (59%)
Hispanic/Latino	6 (19%)
Mixed-Race	5 (16%)
Asian	2 (6%)
Ethnicity	
Spanish, Hispanic, or Latino	13 (41%)
Not Spanish, Hispanic, or Latino	19 (59%)
Primary language	
English	31 (97%)
Spanish	1 (3%)
Highest education obtained	
In high school/did not complete high school	2 (6%)
High school diploma	19 (60%)
Bachelor’s degree	6 (19%)
Graduate degree or higher	2 (6%)
Trade or technical training	2 (6%)
Other	1 (3%)
Employment status	
Employed full time (35+ hours/week)	7 (22%)
Employed part time (less than 35 h/week)	4 (13%)
Full-time student	2 (6%)
Not employed or in school	18 (56%)
Declined to answer	1 (3%)
Opioid treatment status	
Currently in treatment	28 (88%)
Not currently in treatment	2 (6%)
Declined to answer	2 (6%)
Treatment modality	
Outpatient	27 (84%)
Not currently in treatment	2 (6%)
Declined to answer	2 (6%)
Detox	1 (3%)
Medication for opioid use disorder	
Methadone	14 (44%)
Buprenorphine/suboxone	14 (44%)
No medication	3 (9%)
Declined to answer	1 (3%)

### Data collection

An interview guide was developed in meetings and in collaboration with the first four authors of this article. We started by compiling the broad categories relevant to the research questions, then decided on the order of questions and finished by creating probing questions. Interviews took place virtually using Zoom with enhanced security and privacy protections. For participants who did not have access to a device with audio and video they were able to connect to Zoom by calling a phone number provided *via* Zoom’s audio only feature. Every attempt was made to accommodate the schedules of participants. Once the interview was scheduled, the research associate sent participants an invitation containing the meeting details. The email message recommended that participants have their video on during the interview. Before the discussion, the interviewer or research associate reviewed the ground rules, which summarized components of the consent (e.g. maintaining confidentiality, welcoming honest feedback, ability to withdraw, use of audio recording and ability to skip questions). All participants provided verbal consent prior to the audio recording and the start of the interview questions. Participants were administered a short demographic survey by the interviewer or research staff. All of the interviewers had qualitative experience and worked with SUD populations extensively. Using the interview guide, an experienced qualitative interviewer asked participants questions on the impact of COVID-19 as it relates to substance use as well as vaccination perceptions. Questions related to substance use focussed on substance use before, during and after the patient’s treatment episode. Interviews lasted between 45 and 70 min and participants received $50 for their participation. The interviews were recorded using a handheld recorder or a computer application to avoid automatic capture of participants’ faces in Zoom’s built-in recorder. Data collection continued until data saturation was achieved. Data saturation was assessed by the research team at least monthly and when no new information relating to the research questions were obtained no new interviews were scheduled [32]. The institutional review board at UCLA approved all materials and procedures.

### Data analyses

Audio recordings of the interviews were transcribed verbatim by a third party and identifying information was removed. Once data collection efforts were underway all interviewers met twice to discuss findings and update the interview guide as needed. For instance, the first and only significant revision to the interview guide was to ask more questions about the relationship between substance use and risk of contracting COVID-19 as this theme emerged as important during the first few interviews. Once interviews were completed, an initial content analysis using a directed approach was conducted to identify and describe themes [33]. Using the interview guide, three authors developed the first draft of the code list. Two authors then independently reviewed and coded all transcripts using Microsoft Excel [34]. The two authors met several times during the coding process to arrive at a consensus on the final coding scheme. Then, the two researchers reviewed the final list of codes together and discussed any disagreements to reach a consensus [35]. Once themes were identified and described the two researchers met again to further organize themes and identify quotes for the manuscript. The qualitative data analysis and the results reporting were guided by COREQ [36].

## Results

Participants were predominately male (66%), white (59%), had a high school diploma (60%), were either unemployed or in school (56%), were currently in treatment (88%) and were in treatment at an outpatient facility (78%; Table 1). Three primary themes were identified: (1) perceptions and beliefs about COVID-19 susceptibility and severity; (2) perceptions of COVID-19 risk compared to risks associated with substance use behaviours; and (3) vaccine hesitancy.

### Perceptions and beliefs about COVID-19 susceptibility and severity

Participants in our study were mixed in their beliefs of susceptibility to contracting COVID-19 and the severity of the disease if contracted. For example, one participant was concerned about the virus but did not start ‘caring’ until they obtained substance-free public housing for unhoused populations. When asked why the participant became more concerned about COVID-19 over time, they reported, ‘I was concerned about it, but I was in a place at that time where I didn’t really care if I died or anything’. When probed, the participant elaborated,

Once I had a place to live, then it got easy. It got easier and I started caring about stuff again. I didn’t care before, but after I got housing, I started to care again. Care about my appearance and getting sick and stuff like that. (Male; participant #180)

Several participants did not feel that COVID-19 was a serious disease. ‘COVID’s just another thing. It’s just another, like I said, flu. It’s just one of those things that comes and goes. You really don’t pay attention to none of that when you’re using. None of that matters’ (Female; participant #72). Others became cautious about COVID-19 after learning more facts about the disease or having close friends or family members who had contracted it. One participant noted,

I was never concerned. I was never concerned about COVID honestly, at all, until I went to the hospital. You hear a lotta Code Blues coming on, and it’s all related to COVID. Learning about how people die from it, and it sounds scary. That’s when I started becoming like really aware of it and more cautious, I guess. (Male; participant #179)

### Perce*ptions of COVID-19 risk compared with risks associated with substance use*

Many of the participants commented on the risk associated with substance use compared to the risks associated with COVID-19. As one participant stated, ‘Probably the using is more risky than the COVID because you can’t overdose on COVID’ (Male; participant #184). Some participants made statements that the virus was not a concern to them because of the risk they take, or have taken, to use substances which they perceived as a greater risk than acquiring COVID-19. For instance, one participant noted, ‘I already had taken risk before when I was using. I guess I probably just—the reward in my mind was worth the risk, and so I just treated it like normal’ (Male; participant #89).

Some of the participants did not take COVID-19 precautions when buying drugs. As one participant explained, ‘If someone has to get—yeah, if someone wants heroin, they have to go to someone’s house that has COVID. They don’t care. They’re goin’ there to get it as long as they get it’ (Male; participant #195). Likewise, a participant contrasted injecting drugs with wearing a mask to underscore their perceptions on the risk differences between substance use behaviours and COVID-19 mitigation behaviours, ‘Well I’m about to put a needle in my arm with a substance that very well could kill me, so my last concern is wearing a mask’ (Male; participant #66). Other participants reported that using drugs took priority over COVID-19 mitigation strategies. As one participant put it, ‘We definitely didn’t socially distance, sharing straws with buddies, like the straws we used to smoke the drug with. We’d still share the straws and everything, and not wearing masks in the same room and all that’ (Male; participant #164). Likewise, another participant recounted, ‘When you’re homeless on the street using drugs, you don’t worry about that stuff. You just care about one thing and that’s getting high’ (Male; participant #180). Several participants noted how the risks of contracting the virus were less significant in comparison to the risk of using and accessing substances.

While some of the participants noted that they were not worried about contracting the virus, other participants stated they did not want to contract COVID-19 and took steps to avoid contact with people who could have it when possible. For example, one participant explained their attempts to reduce their risk, ‘Socially distance, I would try, but you have to make some sort of contact to switch hand-to-hand transactions’ (Female; participant #45). Participants also stated that they sometimes would have to interact with people in order to buy drugs despite their discomfort in a COVID-19 context. As one participant recounted, ‘Ain’t nobody trying to do a transaction with the—just open face. Somebody’s going just, you know?……Paranoia, be in prayer, you don’t want nobody see your face, the transaction’ (Male; participant #173).

### Vaccine hesitancy

Most of the participants (23 out of 32) reported that they had received the COVID-19 vaccine but 19 of the 32 stated they were hesitant to obtain the vaccine. Among the 9 participants who did not get vaccinated, 7 of the 9 stated they were hesitant to get vaccinated, and among the 23 who had received the vaccine, 12 stated they were hesitant to either get the vaccine or the booster dose. As one participant noted, ‘My concern about the booster shot, I don’t know. Even though I'm a heroin addict I don’t want anybody fillin’ me up with anymore drugs I guess. That’s why I didn’t get my booster shot’ (Male; participant #195).

Only two participants indicated transportation issues were obstacles to obtaining the vaccine. Most participants in the study noted that their hesitation was due to their perception that (1) the vaccine development was rushed and thus unsafe or suspicious, (2) concerns that the government was implanting a microchip in the vaccine and (3) the vaccine was used by the government to control people or that COVID-19 was developed and purposefully released to control people. For example, a participant expressed concerns about the vaccine’s safety and recentness,

Just because it’s not been around long enough at all. I feel like that I wouldn’t be comfortable until it’s been [sighs] maybe a minimum of at least five years. I feel like right now people are so guinea pigs. (Female; participant #171)

Another participant expressed concerns about unknown long-term effects of the vaccine, equating the risks of the vaccine to acquiring mesothelioma. ‘I don’t think it’s really been tested properly. You always have those mesothelioma commercials that pop up a couple of years after the fact and stuff like that, so I just don’t trust it’ (Female; participant #95). Another participant noted concerns about lack of pharmaceutical accountability.

These vaccines that they’ve been pushing so hard for everybody to get, do they really know what it—you have to put your trust in these people 100 percent and these same people are making sure that they have no liability before they even get going. (Male; participant #190)

One participant did not want the vaccine because he did not like that it was mandatory and felt that it was discriminatory. This male participant noted,

There’s a lot of people that are very strong about the vaccine. They don’t want it, or I don’t feel that I should have to get it. The only way that I would get it is because I couldn’t go travel or something. As it is, they won’t let me in restaurants or play. I can’t go see plays without a card. It’s really discriminatory. It’s like the new black slave or something, you’re being excluded and bullied pretty much. You’re pretty much being bullied. You can’t go here, you can’t go there unless you have this. (Male; participant #190)

Some participants were initially hesitant to get the vaccine but later received the vaccine. For instance, one participant reported that they were initially hesitant because they worried that the government was trying to control people with the vaccines, but this perception changed once they stopped using drugs.

I thought about it for a second. I don’t know. I read books and they tripped me out but I think it was because I was high. I thought that it was maybe a microchip or something like that. I was like no, they are doing it to follow us and track us and blah blah, but once I got sober it was like go do it and I just got it. It was easy. (Male; participant #179)

Similarly, another participant, who had not received the vaccine, noted that although he initially distrusted the legitimacy of the vaccine, his openness to receiving the vaccine shifted once he was in recovery (quote not shown) and once he learned more about the vaccine and understood misinformation about it.

Yeah, ‘cause I was reading a lot of stuff about it. There’s some sites saying the government was—planted that virus for population control or something. I started readin’ about that for a while and it turned out to be bogus. (Male; participant #180)

Other participants had a concern about how the vaccine could react with their MOUD. ‘I do have many concerns with that, how it’s gonna react with the methadone’ (Female; participant #201). One participant expressed concerns that his health had worsened after obtaining the vaccination.

The first one I was okay, the second one, I was deathly sick for about seven to nine days, laying in bed, shaking, and shivering. They’re talking about a booster shot, I ain’t getting no booster shot after that. Here’s the sad part about it too, is that while I was in custody—I got hepatitis C. While I was in custody, my liver was checked on a regular basis and my liver was fine. I get out, I get the two shots and all of a sudden I got cirrhosis of the liver. Now, I'm wondering if that [COVID-19 vaccine] expedited liver damage for me or what. (Male; participant #88)

## Discussion

Participants expressed diverse perceptions of the seriousness of COVID-19, with some taking precautions to mitigate their chances of acquiring COVID-19 and others perceiving that the risk of contracting COVID-19 was less than the risk of overdosing. Although some participants obtained the COVID-19 vaccine, most participants (19 of 32) reported vaccine hesitancy, noting concerns about long-term safety and misconceptions about the vaccine. These results may point to mistrust of institutional systems exacerbated by stigma and trauma that is often experienced by people with OUD.

Our results revealed that COVID-19 mitigation measures among persons with OUD are influenced by their risk environment that includes social and physical places where different factors interact to increase the chances of harms, especially drug-related harms [37]. These risk environments can shape perceptions of COVID-19 susceptibility and severity and potentially increase risk of exposure to and potential infection with COVID-19. The known dangers of opioid use, as demonstrated by increased overdose deaths in the United States [38,39], understandably could reduce relative perceptions of COVID-19 severity among some of the study participants. Opioid use has become riskier in recent years in part because of the increased presence of fentanyl in illicit opioids, including heroin and tablets made to appear like opioid medications [40] and these dangers related to opioid use may be perceived as greater than the risks of COVID-19.

Some participants stated they took precautions to avoid putting themselves at risk of exposure to COVID-19, but they also noted that they had to interact with others to buy drugs. Although the relationship between substance use and risk of contracting COVID-19 is complex, people with OUD would likely benefit from COVID-19 harm reduction approaches. For example, it is well accepted that patient-centred care using a harm reduction approach reduces the consequences associated with substance use even if complete abstinence is not attained [41,42]. This approach could be applied to reduce COVID-19 harms. Providing masks, COVID-19 testing kits and COVID vaccinations at SUD harm reduction and treatment sites are just a few of the methods that could be helpful for persons with OUD. Additionally, providers could routinely discuss the importance of hand washing, vaccinations, safe supplies and overdose mitigation (e.g. naloxone) with their patients in a non-judgmental manner. Combining the discussion of COVID-19 precautions with overdose prevention would provide a person-centred approach to healthcare that would likely increase health and wellbeing among persons with OUD.

Concerns about vaccine safety were the leading reason for vaccine hesitancy in our sample and are consistent with previous quantitative studies on vaccine intentions among the general population [43]. While COVID-19 vaccination acceptance in the U.S. among the general population appears to be increasing with time [44,45] it is unknown if this trend will occur for people with OUD. A few participants also reported inconvenience as a reason for not getting the vaccine; among vaccine-motivated persons with OUD, low perceived risk of COVID-19 and inconvenience (e.g. geographical difficulties to access, visiting centre schedule) have been found to be the main barriers to vaccination [46]. Reducing barriers to care by providing rideshare vouchers and efforts to overcome vaccine misinformation to increase vaccination rates should be prioritized.

Some of our participants considered COVID as a secondary concern related to their health but this is a misconception because patients with SUD are at a higher risk of serious, life-threatening complications from COVID-19. There is an increased risk of COVID-19 severity among people with SUDs, and this severity is pronounced among people with OUD [11,47]. Vaccinated people with SUDs are also at higher risk for breakthrough COVID-19 infection [12]. Therefore, vaccination and treatment of COVID should be prioritized among patients with SUDs, and integrated care is recommended, including COVID vaccination in addiction medicine settings and flexible MOUD (such as home delivery, no contact dosing) for patients who are COVID positive [48,49].

For people with OUD, the immediate fear of physical discomfort from being without opioids (e.g. withdrawal and craving) may outweigh concern about getting sick from COVID-19 at a later time point. Access to low-barrier buprenorphine and telemedicine-based care is vital to addressing the deleterious interaction of the two crises. Approved medications, including buprenorphine and methadone, are well-established, effective treatments for OUD [50,51] that not only reduce overdose deaths but have been shown to decrease all-cause mortality [52]. Furthermore, treatment with buprenorphine or methadone is associated with a reduction in opioid overdose and serious acute care when compared with other forms of treatment such as residential treatment, intensive behavioural health treatment, naltrexone treatment, or non-intensive behavioural health treatment [51]. Low-barrier MOUD and reducing barriers to MOUD [53] (e.g. mobile clinics, tele-prescribing) have been useful throughout the pandemic.

Telemedicine and low-barrier MOUD have been used throughout the pandemic to reduce the negative impact the pandemic has had on patients with OUD and to increase buprenorphine prescribing [3,54]. It appears that less restrictive buprenorphine prescribing guidelines have led to increased access to buprenorphine [55] and improved retention in care [56]. Some have argued that the temporary changes in restrictions on MOUD prescribing should be made permanent so that vulnerable individuals with OUD do not face excess barriers to care [57]. Overdose death rates increased sharply during the pandemic, COVID-19 comorbidity is higher among OUD populations, substance use seems to reduce efforts at COVID-19 mitigation for some, and concerns about vaccine interaction with MOUD make a strong case for why increasing access to MOUD with low-barrier methods should be prioritized.

## Limitations

Several study limitations warrant mention. First, participants had been in MOUD treatment at various times during the initial pandemic onset and during the ongoing pandemic (from August 2020 to March 2022). Second, while the parent study may have increased participation among patients who experience transportation barriers to a research office, the use of a virtual data collection modality potentially limited individuals who did not have access to the internet or internet-equipped mobile phones or computer devices. Third, the impact of COVID-19 may be more severe on those individuals who used illicit substances and were not in treatment, were unhoused, incarcerated, undocumented, or from another vulnerable populations. Those voices are not reflected in the current study and thus the perceptions expressed in this study may differ from those who were unable or unwilling to participate. Future research should examine how to best reach the most vulnerable populations to examine the impact of COVID-19 on substance use behaviours and vaccine hesitancy. Despite these limitations, our study provides insight into the relationships between substance use behaviours that could increase COVID-19 infection in a sample of patients receiving (or previously receiving) MOUD.

## Conclusions

Some of our participants considered COVID as a secondary concern related to their health in part because of their need to obtain substances, underscoring the importance of providing a broad range of treatment options for persons with OUD that support harm reduction and recovery. Access to regular COVID-19 testing, treatment and vaccinations should be prioritized for this vulnerable group as OUD is a condition that increases the risk for COVID-19. Our findings provide context to the barriers to better health for persons with OUD that could be useful for providers trying to help their patients make informed decisions on their health, such as getting vaccinated and engaging in MOUD.

Public health measures aimed at the general public may unintentionally increase other risks for people living with addictions. In the case of COVID-19, social distancing measures for people who use substances could increase overdose risk for some individuals. For future health pandemics beyond COVID-19, public health measures will need to be adapted for people with OUD/SUD, as well as patients with other chronic conditions. Public health strategies should include harm reduction for both substance use and public health measures. Reducing risk rather than eliminating of risk should be a focus for public health workers and medical providers. Public health strategies should focus on transparent communication about the risks/benefits of various measures to control disease, with an understanding of patients’ individualized concerns and service needs and tailor strategies to ensure continued access to care among vulnerable populations (e.g. MOUD and harm reduction).

## Data Availability

The data used in this research are not publicly available due to concerns about confidentiality. However, we have summarized our participants and provided descriptive quotes in the manuscript text. The data that support the findings of our qualitative study are available upon reasonable request.
